# Historical BCG vaccination combined with drug treatment enhances inhibition of mycobacterial growth *ex vivo in human peripheral blood cells*

**DOI:** 10.1038/s41598-019-41008-4

**Published:** 2019-03-19

**Authors:** Satria A. Prabowo, Andrea Zelmer, Lisa Stockdale, Utkarsh Ojha, Steven G. Smith, Karin Seifert, Helen A. Fletcher

**Affiliations:** 1 Department of Immunology and Infection, Faculty of Infectious and Tropical Diseases, London School of Hygiene and Tropical Medicine, London, UK; 2 Tuberculosis Centre, London School of Hygiene and Tropical Medicine, London, UK; 3 Faculty of Medicine, Imperial College School of Medicine, Imperial College London, London, UK

## Abstract

Tuberculosis (TB) is a leading infectious cause of death globally. Drug treatment and vaccination, in particular with Bacillus Calmette-Guérin (BCG), remain the main strategies to control TB. With the emergence of drug resistance, it has been proposed that a combination of TB vaccination with pharmacological treatment may provide a greater therapeutic value. We implemented an *ex vivo* mycobacterial growth inhibition assay (MGIA) to discriminate vaccine responses in historically BCG-vaccinated human volunteers and to assess the contribution of vaccine-mediated immune response towards the killing effect of mycobacteria in the presence of the antibiotics isoniazid (INH) and rifampicin (RIF), in an attempt to develop the assay as a screening tool for therapeutic TB vaccines. BCG vaccination significantly enhanced the ability of INH to control mycobacterial growth *ex vivo*. The BCG-vaccinated group displayed a higher production of IFN-γ and IP-10 when peripheral blood mononuclear cells (PBMC) were co-cultured with INH, with a similar trend during co-culture with RIF. A higher frequency of IFN-γ^+^ and TNF-α^+^ CD3^−^ CD4^−^ CD8^−^ cells was observed, suggesting the contribution of Natural Killer (NK) cells in the combined effect between BCG vaccination and INH. Taken together, our data indicate the efficacy of INH can be augmented following historical BCG vaccination, which support findings from previous observational and animal studies.

## Introduction

Tuberculosis (TB) is a leading infectious cause of death worldwide. Over the past 200 years, the disease has killed one billion people, surpassing any other infectious disease^1^. In 2016, it is estimated that TB affected 10.4 million people and killed 1.7 million individuals^2^. The WHO “End TB” strategy aims to end the global TB epidemic in 2035 by reducing TB incidence by up to 90% and deaths by 95%^3^. Optimising the use of current, and developing new tools are essential to achieve the desired goals.

Drug treatment and vaccination remain the main strategies currently being used to control the TB epidemic caused by *Mycobacterium tuberculosis* (*Mtb*). The current treatment regimen for drug-sensitive TB lasts for 6 months and consists of several first-line drugs. Although the regimen provides 95% cure rates for drug-sensitive TB^4^, it is still considered lengthy and has led to poor adherence for patients in many settings^5^. Moreover, the emergence of multi-drug resistant (MDR)-TB, which is defined as resistance towards isoniazid (INH) and rifampicin (RIF) – the two major first-line TB drugs – has challenged the effectiveness of chemotherapy in the future^6^.

Bacille Calmette-Guérin (BCG), a live attenuated strain of *Mycobacterium bovis*, is the only vaccine licensed for TB. BCG is widely used to prevent TB in children since the 1970s as an important part of the Expanded Program on Immunization and has since been given more than 4 billion times^7^. BCG is known to be 80% protective in the UK, although the vaccine is considered to have a variable efficacy in countries closer to the equator towards the prevention of pulmonary TB in adults^8,9^. Combining TB vaccination with drug treatment has been proposed to shorten treatment duration and prevent relapse, an approach known as therapeutic vaccination^10,11^. An early animal study by Dhillon and Mitchison (1989) demonstrated the beneficial effect of drug therapy combined with previous BCG vaccination in the guinea pig^12^. In a more recent study by Shang *et al*. (2012), prior BCG vaccination as an adjunct to chemotherapy significantly improved survival of guinea pigs challenged with a virulent strain of *Mtb*^13^.

A therapeutic vaccination strategy for TB is expected to provide benefits in the context of treatment for both active and latent TB. Evidence from leprosy, a disease caused by the same genus of mycobacteria, demonstrates the synergistic effect between historical BCG vaccination and rifampicin prophylaxis treatment for the disease, increasing treatment efficacy up to 80%^14,15^. Despite this, there are few studies which attempt to administer TB vaccines therapeutically after infection for enhancement of TB drug efficacy. This is partly due to the historical experience with tuberculin, a crude extract of *Mtb*, which resulted in an exacerbated immune response when administered therapeutically in active TB patients (known as the “Koch phenomenon”). However, at that time tuberculin was administered alone due to the lack of treatment options. Recently, it was shown that the occurrence of such exacerbated response is associated with bacterial load^16^. Therefore, it is suggested that a therapeutic TB vaccine needs to be administered following chemotherapy after the bacterial load has been sufficiently reduced, hence ensuring safety and allowing a beneficial synergistic effect^7^.

In order for a novel TB vaccination strategy to be implemented, its efficacy needs to be demonstrated in large and expensive efficacy trials^17^. Recently, funders have become more reluctant to provide the required investment due to the risk of failure^18^. As there are currently at least a dozen of TB vaccine candidates in the clinical trial pipeline and more candidates under pre-clinical development^19^, an effort to screen these candidates at early phases is needed to prioritise which candidates should be tested with the available funding and field sites. In the context of therapeutic vaccination, a screening effort will be essential to narrow down the optimum vaccine and drug regimen before progressing to larger clinical trials.

In this study, we implemented an *ex vivo* mycobacterial growth inhibition assay (MGIA) to measure vaccine-induced bacterial growth inhibition in combination with drug treatment following historical BCG vaccination in healthy human volunteers. In the absence of an immune correlate of protection following TB vaccination, the use of MGIA as a functional assay has gained much interest recently for its ability to assess the cumulative effect of multiple immune components on the control of mycobacterial growth^20–22^. Various cell types are known to play roles in protection against TB, such as lymphocytes, macrophages, dendritic cells and natural killer (NK) cells^23^. Here, we present the findings of our study which evaluates the ability of the MGIA to discriminate the impact of historical BCG vaccination towards the *ex vivo* killing effect of mycobacteria in the presence of the antibiotics INH and RIF. This study, which provides proof-of-principle of the potential of the MGIA to measure a synergistic effect between vaccination and chemotherapy, is important as the MGIA can be used to further expedite the development of therapeutic vaccination strategies for TB. Our study shows the ability of the MGIA to capture vaccine mediated growth inhibition, even in the presence of effective drugs that substantially reduce mycobacterial growth, and supports the implementation of the MGIA as a screening tool for therapeutic TB vaccine candidates.

## Results

### Study participants

Fifty participants were enrolled in the main cohort of this study; 21 vaccine-naïve volunteers with no history of BCG vaccination and 29 volunteers previously vaccinated with BCG (average time since vaccination 25.4 years prior to enrolment). Table 1 summarises the demographic characteristics of the study participants. Almost 70% of the BCG-vaccinated participants were from the UK.

**Table 1:** Demographics of participants enrolled in the study.

Characteristic	Main Cohort (Total Participants: 50)		Alternate Cohort (Total Participants: 50)	
	BCG-naïve (n = 21)	BCG-vaccinated (n = 29)	BCG-naïve (n = 16)	BCG-vaccinated (n = 34)
Female [no. (%)]	17 (81.0%)	15 (51.7%)	11 (68.75%)	27 (79.4%)
Median age [yr (range)]	30 (22–69)	33 (22–63)	27.5 (23–53)	33.5 (25–70)
Average time since BCG vaccination [yr (range)]	—	25.4 (9–52)	—	25.9 (10–48)
Country of Origin UK [no. (%)]	5 (23.8%)	20 (69%)	3 (18.8%)	24 (70.6%)

### IFN-γ ELISpot response to PPD and assessment of *ex vivo* growth inhibition

**Figure 1:**
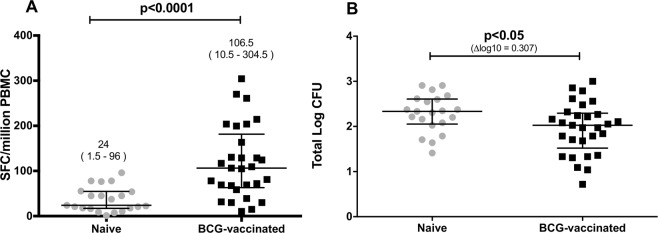
Immune response (**A**) and growth inhibition (**B**) following historical BCG vaccination. Assessment was performed from 21 BCG-naïve and 29 BCG-vaccinated participants. (**A**) IFN-γ production following stimulation with PPD was compared (Mann-Whitney test). Numbers above each group represent median (range). SFC, spot forming cells. (**B**) Growth inhibition was compared using BCG input ∼100 Colony Forming Unit (CFU) as immune target (unpaired t-test). Data are presented as total number of log CFUs per sample, which was determined by use of a standard curve. Dots and squares represent individual data points, and the central lines indicate the median response with inter-quartile range (IQR).

An Interferon-γ (IFN-γ) Enzyme-Linked ImmunoSpot (ELISpot) assay was performed to measure the magnitude of mycobacteria-specific response from historically BCG-vaccinated and BCG-naïve participants. The secretion of IFN-γ in response to purified protein derivate (PPD) was elevated in the samples from vaccinated individuals in comparison to unvaccinated individuals (median SFC 106.5 and 24, *P* < 0.0001, Fig. 1A). A growth inhibition assay was performed to assess impact of BCG vaccination on *ex vivo* mycobacterial growth. The assay was termed ‘growth inhibition’ as the immune responses in the vaccinated group are expected to inhibit the growth of mycobacteria compared to the naïve group during the 4-days co-culture. Using cryopreserved Peripheral Blood Mononuclear Cells (PBMCs), the assay showed enhanced mycobacterial growth inhibition in PBMCs from BCG-vaccinated compared to BCG-naïve individuals (median log CFU 2.027 and 2.334, *P* < 0.05, Fig. 1B). There was no statistically significant correlation between IFN-γ ELISpot response and mycobacterial growth (*P* = 0.121, Spearman r = −0.22, data not shown).

### Drug titration curves and impact of historical BCG vaccination on drug-mediated *ex vivo* growth inhibition

**Figure 2:**
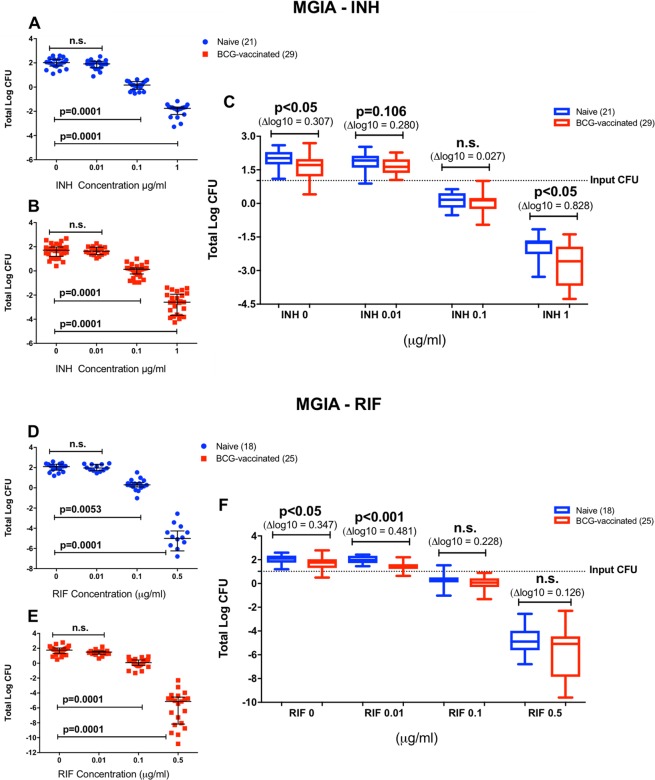
Growth inhibition in the absence and presence of INH (**A**) and RIF (**B**). Mycobacterial growth was assessed in titration curves. INH inhibited mycobacterial growth in a dose-dependent manner in the naïve and vaccinated groups (**A**,**B**), as well as RIF (**D**,**E**). Data from both groups was compiled in dose-response box plots to identify the BCG effect in addition to the INH- and RIF-mediated killing (**C**,**F**). Dots and squares in the titration curves (**A**,**B**,**D**,**E**) represent individual data points from the participants and the central lines indicate the median response with IQR. Each group is represented in a single box plot with range in the dose-response analysis (C and F). Samples size is indicated in the figure (for INH: n = 29 BCG-vaccinated and n = 21 BCG-naïve participants; for RIF: n = 25 BCG-vaccinated and n = 18 BCG-naïve participants). Statistical significances were tested using one-way ANOVA (**A**,**B**,**D**,**E**) and unpaired t-test (**C**,**F**).

Drug concentrations were selected to achieve a concentration range where we could observe a decrease in bacterial growth, but sufficient bacterial load to identify any synergistic effect of vaccination in addition to the drug. Previous studies have identified the minimum inhibitory concentration (MIC) of INH and RIF towards BCG Pasteur to be 0.1 and 0.063–0.125 µg/ml, respectively^24^. The drug concentration of 0.1 µg/ml was chosen as it closely depicts the MIC value of INH and RIF. Concentrations were then selected above and below that to assess potential synergistic effects. As expected, there were significant reductions of bacterial growth when BCG was co-cultured with PBMC and 0.1 and 1 µg/ml of INH (*P* = 0.0001, Fig. 2A,B), as well as 0.1 and 0.5 µg/ml of RIF (*P* = 0.0053 and *P* = 0.0001, Fig. 2D,E) in both BCG-naïve and vaccinated groups. Findings in this study were consistent with previously published results regarding the MIC and drug susceptibility of BCG Pasteur^24^.

To determine if historical BCG vaccination enhanced the drug effect, data from each BCG-naïve and vaccinated group were plotted in dose response box plots to observe the vaccine impact at various drug concentrations (Fig. 2C,F). BCG vaccination significantly enhanced the ability of INH to control mycobacterial growth at the concentration of 1 µg/ml (*P* < 0.05), with no significant differences at INH concentrations of 0.01 and 0.1 µg/ml (*P* = 0.106 and 0.469, respectively, Fig. 2C). The effect size of growth inhibition at INH concentration of 1 µg/ml was greater (0.8 log) compared to at the absence of drug (0.3 log). Meanwhile, BCG vaccination did not enhance the control of mycobacterial growth at RIF concentrations of 0.1 and 0.5 µg/ml and the difference was only statistically significant at 0.01 µg/ml (Fig. 2F). The slope of the titration curve was steeper with RIF compared to INH even though a lower maximum concentration of RIF was used (0.5 µg/ml). The log CFU values were obtained by converting the recorded time to positivity (TTP) of the Bactec MGIT 960 used for quantification using a standard curve (see Supplementary Fig. S1). Negative log CFU values indicate low growth of mycobacteria which were still detected using the MGIT machine and extrapolated using the standard curve.

Similar observations were obtained when the MGIA was performed with mouse splenocytes vaccinated with BCG at a determined time point (six weeks before sacrifice). Protocol from a previously published study^25^ was adopted to assess the impact of BCG vaccination on the killing of INH and RIF in mice, as performed with human PBMCs in this study. BCG vaccination in mice significantly enhanced the ability of INH to control mycobacterial growth at the concentration of 1 µg/ml (*P* < 0.05) (Supplementary Fig. S2A). Meanwhile, BCG vaccination did not enhance the control of mycobacterial growth at any RIF concentrations (Supplementary Fig. S2B). Similar titration curves were observed with INH and RIF during co-culture with mouse splenocytes compared to human PBMCs (Supplementary Fig. S2C–F).

### Cytokine release associated with *ex vivo* growth inhibition

**Figure 3:**
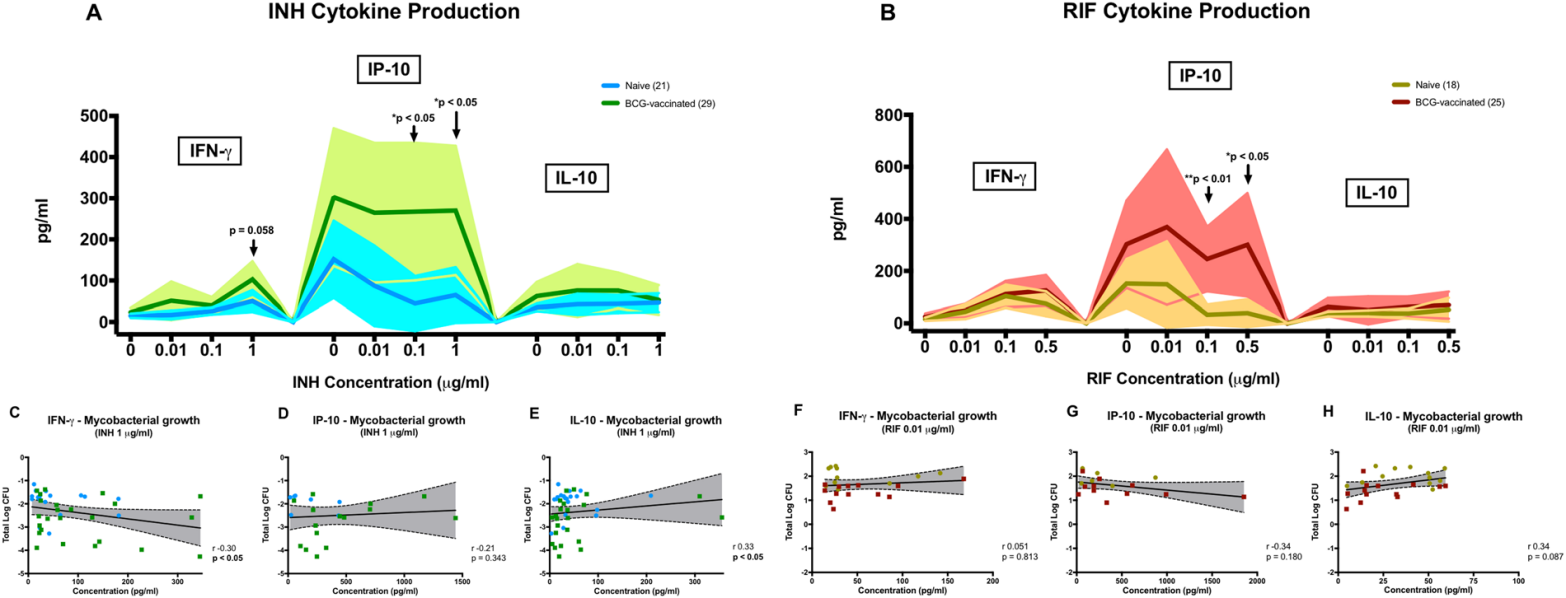
Cytokine responses from co-culture with INH (**A**) and RIF (**B**). MGIA supernatants were analysed for the released cytokines IFN-γ, IP-10 and IL-10. For INH, dark blue and dark green lines and symbols indicate BCG-naïve and BCG-vaccinated groups, respectively. For RIF, these were represented by dark brown (BCG-naïve) and dark red (BCG-vaccinated). Lines indicate mean response and shadings indicate range (**A**,**B**). Comparison of responses between BCG-vaccinated and BCG-naïve groups at different drug concentrations were performed using unpaired *t*-test. Correlation between mycobacterial growth at INH concentration of 1 µg/ml and the production of IFN-γ (**C**), IP-10 (**D**) and IL-10 (**E**) were assessed using Spearman’s correlation. Correlation between mycobacterial growth at RIF concentration of 0.01 µg/ml (based on the significant difference in the MGIA assay) and the production of IFN-γ (**F**), IP-10 (**G**) and IL-10 (**H**) were also assessed (Spearman’s). Note: for the correlations, non-responders were excluded, defined as responses below the following cut-off of the ELISA assays: 7.5 pg/ml (IFN-γ), 5 pg/ml (IP-10) and 3.5 pg/ml (IL-10). Refer to Supplementary Table S1 for comparison and correlation of other cytokines responses.

ELISA assays were performed using the MGIA supernatant to investigate cytokine productions (IFN-γ, IP-10, IL-10, TNF-α, IL-12p40, GM-CSF, IL-6 and IL-17) which may be associated with *ex vivo* growth inhibition at all drug concentrations. A higher production of IP-10 was observed in the BCG-vaccinated group compared to the BCG-naïve group when PBMC were co-cultured with 1 µg/ml of INH (*P* < 0.05, Fig. 3A), with a similar non-significant increase of IFN-γ (*P* = 0.058). At this drug concentration, there was a significant moderate inverse correlation between IFN-γ production and *ex vivo* growth inhibition (Spearman r = − 0.30, *P* < 0.05, Fig. 3C). Notably, this was where we observed a significant difference in the MGIA assay. A significant increase of IP-10 was also observed in the BCG-vaccinated group at INH concentration of 0.1 µg/ml (Fig. 3A). Moreover, there was a significant moderate positive correlation between IL-10 production and higher growth of mycobacteria at 1 µg/ml INH (Spearman r = 0.33, *P* < 0.05, Fig. 3E), although we did not see significant differences of IL-10 production between vaccination groups at various INH concentrations (Fig. 3A). Other cytokines measured using co-culture supernatants with INH are summarised in Supplementary Table S1. In general, there appears to be higher cytokine productions in the historically BCG-vaccinated participants when compared to the BCG-naïve, in the presence and absence of drugs. In this study, we consider the BCG-naïve participants as a control for the responses observed in the BCG-vaccinated participants. Therefore, introducing a non BCG-stimulated condition in the assay was not performed, given the limited cell numbers from our participants.

Similarly to INH, a higher production of IP-10 in the vaccinated group was also observed during the co-culture with RIF at the concentration of 0.1 and 0.5 µg/ml (*P* < 0.05, Fig. 3B), compared to the BCG-naïve group at each drug concentration. There was a significant moderate positive correlation between IL-10 production and higher growth of mycobacteria at a RIF concentration of 0.1 µg/ml (Spearman r = 0.37, *P* < 0.05, Supplementary Table S1), with a non-significant correlation at the concentration of 0.01 µg/ml (Spearman r = 0.34, *P* = 0.087, Fig. 3H). Other cytokines measured using co-culture supernatants with RIF are summarised in Supplementary Table S1.

### Intracellular Staining Flow Cytometry

Intracellular cytokine staining (ICS) of BCG-stimulated PBMCs followed by flow cytometry analysis was performed to detect the ability of historical BCG vaccination to induce cytokine-secreting lymphocytes during the co-culture with drug and BCG. ICS flow cytometry enabled the simultaneous detection of CD4^+^ and CD8^+^ T-cells as well as CD3^+^ CD4^−^ CD8^−^ (double negative = DN) and CD3^−^ CD4^−^ CD8^−^ (triple negative = TN) cells, and secretion of cytokines such as IFN-γ, TNF-α and IL-2 in BCG-stimulated PBMCs (see Supplementary Fig. S3 for gating strategy). The selected drug concentration was 1 μg/ml of INH as the vaccine effect was most notable at this concentration and a significant correlation with cytokine response, in particular with IFN-γ and IP-10, was observed.

**Figure 4:**
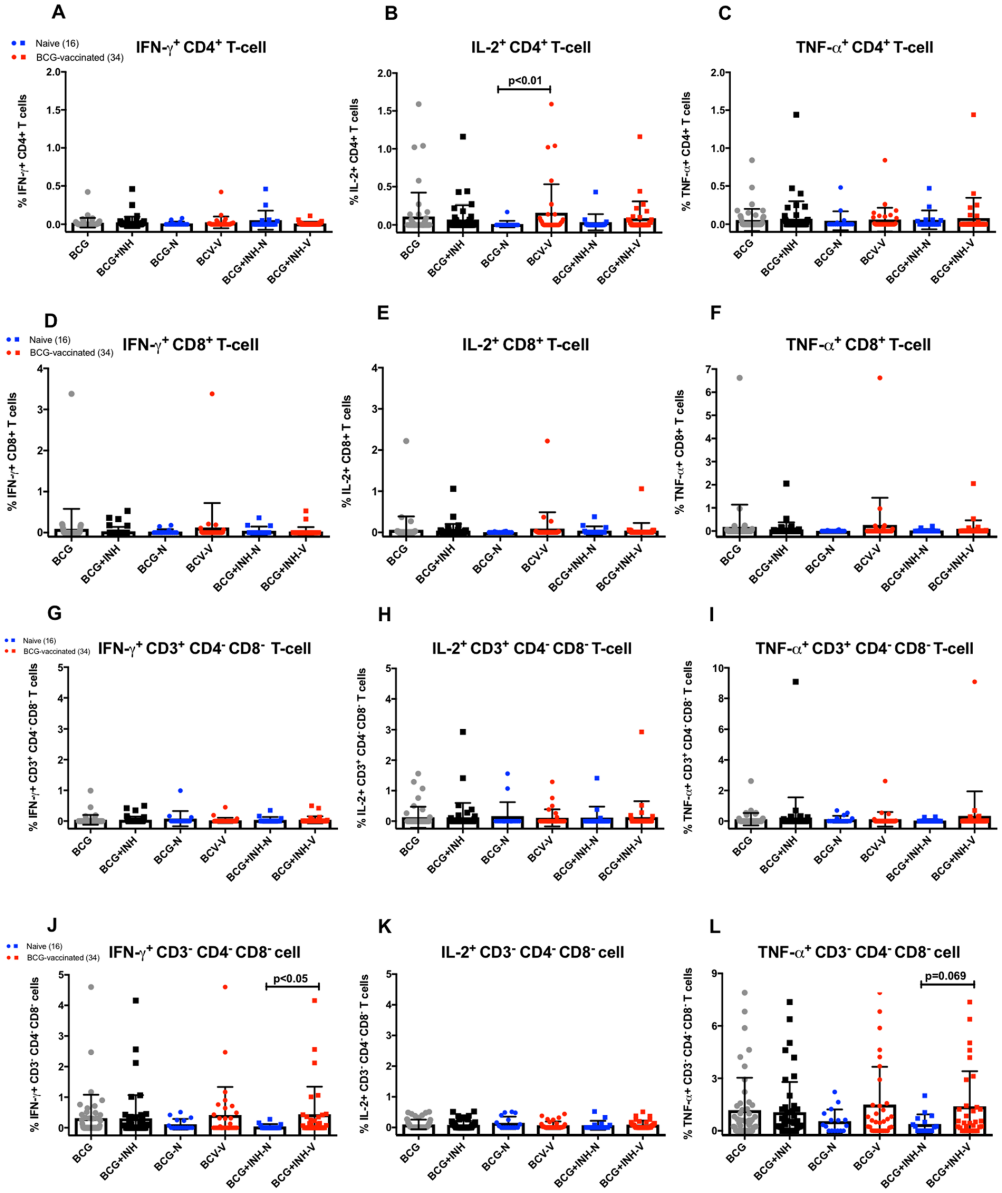
Frequencies of Th1 cytokine-expressing lymphocytes. Expressions were measured from PBMCs after stimulation with BCG, with and without 1 μg/ml of INH, for 4 days. The grey dot symbols represent stimulation with BCG only, while the black squares represent BCG + INH. Comparisons were made between the naïve (blue) and historically BCG-vaccinated (red) groups. Data is presented as percentages of cell population of interest, and displayed as bar graphs with error bars represent mean ± SD. For this experiment, PBMCs from an alternate cohort of participants were used (Table 1), consisted of 16 naïve and 34 historically BCG-vaccinated participants, with a comparable demographics profile and immune responses as the main cohort. The Mann-Whitney *U* test was used to determine significance. The *P* value < 0.05 is considered statistically significant.

Historical BCG vaccination was shown to result in an antigen-specific Th-1 response which was detectable in the ICS assay upon stimulation with BCG bacteria (Fig. 4). There was a significantly higher frequency of BCG-specific CD4^+^ T-cells expressing IL-2 in the historically BCG-vaccinated group compared to the naïve in the absence of drug (mean frequency 0.160% and 0.011%, *P* < 0.01, Fig. 4B). Although statistical significance was not arrived at, there was also slightly higher expressions of IFN-γ (mean frequency 0.01% and 0.0075%) and TNF-α (mean frequency 0.0875% and 0.015%) in the BCG-vaccinated group compared to the naïve (Fig. 4A,C, respectively). Cytokine expression of BCG-specific CD8^+^ T-cells appeared to follow the same pattern, despite the responses were lower compared to the CD4^+^ T-cells and did not reach significance (Fig. 4D,E). The ICS panel used in this experiment allowed us to further look at two other lymphocyte populations: double-negative CD3^+^ CD4^−^ CD8^−^ T-cells, which consist primarily of γδ T-cells and CD4^−^ CD8^−^ TCR-αβ^+^ T-cells, and triple-negative CD3^−^ CD4^−^ CD8^−^ cells which are primarily natural-killer cells^23^. In the absence of drug, the frequencies of TN cells expressing IFN-γ and TNF-α appeared to be higher in the BCG-vaccinated group compared to the naïve, although it did not reach significance (Fig. 4J–L), and no observed difference of the cytokine expression from DN T-cells (Fig. 4G–I).

When the co-culture stimulation was performed in the presence of INH to investigate the source of increased IFN-γ production observed in the ELISA assay, a significantly higher frequency of IFN-γ^+^ triple-negative cells was observed in the BCG-vaccinated group compared to the naïve (mean frequency 0.439% and 0.04%, *P* < 0.05, Fig. 4J), suggesting NK cells as a potential source of IFN-γ which could be enhanced in the presence of drugs *ex vivo*. A slightly higher frequency of TNF-α^+^ TN cells was also observed (mean frequency 1.4% and 0.388%, *P* = 0.069, Fig. 4L). There was no differential cytokine expression from CD4^+^, CD8^+^ and double-negative T-cells during the co-culture with 1 μg/ml of INH, proposing the role of a T-cell independent mechanism on the potential synergistic effect of historical BCG vaccination and drug-mediated growth inhibition which has been observed in our *ex-vivo* MGIA assay.

**Figure 5:**
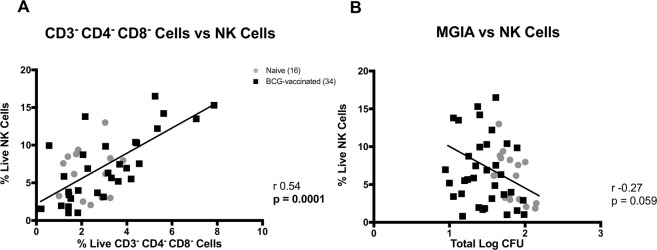
NK cells correlations. The frequency of CD3^−^ CD4^−^ CD8^−^ cells was correlated with NK cells (CD3^−^ CD56^+^) (**A**) and the *ex vivo* mycobacterial growth was associated with the NK cells frequency (Spearman’s correlation) (**B**). Surface-staining flow cytometry was performed to characterise NK cells. PBMCs from 16 naïve and 34 historically BCG-vaccinated participants were used.

As the triple-negative CD3^−^ CD4^−^ CD8^−^ cells identified in this study were thought to be mostly NK cells, surface-staining flow cytometry was performed on unstimulated PBMCs from above cohort using markers for NK cells (CD3^−^ CD56^+^). There was a significant positive correlation between the frequency of CD3^−^ CD4^−^ CD8^−^ cells and the frequency of NK cells (*P* = 0.0001, Fig. 5A). Moreover, a non-significant inverse correlation was observed between the frequency of NK cells and *ex vivo* mycobacterial growth in the absence of drug (*P* = 0.059, Spearman r = −0.27, Fig. 5B).

## Discussion

Participants with historical BCG vaccination in our study were shown to elicit stronger IFN-γ response towards PPD antigen as well as better control of mycobacterial growth *ex vivo*. The average time since BCG vaccination was 25.4 years. Here, most of the BCG-vaccinated participants were from the UK, where BCG vaccination is known to be 80% protective^8^. Recent studies by Mangtani *et al*. provided evidence that protection from BCG in the UK population could last at least up to 20 years^26,27^, which is consistent with the finding of our study. Several studies in other settings also reported detectable protection following BCG vaccination over more than 20 years^28–30^.

There was no significant correlation between IFN-γ production and *ex vivo* control of mycobacterial growth in our study cohort. This indicates that an IFN-γ response is perhaps only in part essential for control of mycobacterial growth. Recently, IFN-γ has been shown to correlate with lower risk of developing TB disease following BCG vaccination^31^, although some studies suggested otherwise^23,32^. In the Fletcher *et al*. study, CD4^+^ T-cell activation was shown to correlate with increased TB risk, suggesting an interplay of immune pathways of risk and protection in the same individual^31^. The IFN-γ based assay has been recommended by a WHO Panel to be used in TB vaccine trials^33^ and our findings support the notion that a functional assay, which measures the summative effect of immune response following vaccination, might be useful in addition to the IFN-γ-based assay.

In this study using human PBMC, INH and RIF as two front-line anti-TB drugs were tested. Both drugs are used for drug-sensitive TB treatment and have been regarded as key for success in current short-course chemotherapy. However, their effectiveness has been challenged by the emergence of MDR-TB and the effort to improve current treatment is indispensable. As BCG is a live, replicating mycobacteria, administration of BCG at the time of treatment may lead to clearance of BCG, thereby preventing the establishment of a vaccine-specific immune response. In this study, however, we investigated the impact of historical BCG vaccination towards mycobacterial growth inhibition in the presence effective anti-mycobacterial drugs. This study serves as a proof-of-principle for testing and screening therapeutic TB vaccine candidates in combination with drugs using the *ex vivo* MGIA system prior or in adjunct to *in vivo* animal or human testing. The nature of the MGIA system does not require the immune mechanism essential for *ex vivo* control of mycobacterial growth to be known *a priori*, while in turn it could help to identify mechanism by investigating samples with efficient growth inhibition. For this purpose, a TB vaccine still needs to be given to healthy human volunteers. However, the sample size can be substantially reduced as the interaction between different vaccine and drug combinations can be assessed *in vitro*. This would particularly be useful when there is a need to test different vaccine, adjuvant or drug combinations and doses. The MGIA will generate a snapshot of the ability of the immune system to control mycobacterial growth following vaccination in the presence of drug *ex vivo*, which could provide valuable information before progressing to larger human trials.

In the present study, historical BCG vaccination was shown to enhance the ability of PBMC to inhibit mycobacterial growth in the absence of drugs and to further enhance the efficacy of INH at the concentration of 1 µg/ml. Interestingly, this concentration is close to the therapeutic level of INH in the plasma during treatment in human, which ranges from 2–5 µg/ml^34,35^. As the effect size of the growth inhibition was greater during the co-culture with 1 µg/ml INH compared to the absence of drug, we believe this reflects a specific synergistic effect of historical BCG vaccination with INH. We did not observe any impact of historical BCG vaccination at the higher concentration of RIF. Rifampicin has been known to be very potent *in vitro*^36^, as demonstrated in our *ex vivo* system at the tested RIF concentration (0.1 and 0.5 µg/ml) and reflected in the steeper dose-response curve compared to INH. Therefore, as RIF was highly effective we could not observe any impact of historical BCG vaccination on RIF efficacy, despite the increased cytokine production observed in the ELISA assay. These results were further supported and consistent with our findings from the mouse study, in which BCG vaccination given at a determined time point (6 weeks prior to sacrifice) resulted in a similar enhanced killing of INH *ex vivo*, with no notable effect on RIF.

The enhancement of *ex vivo* INH killing effect with BCG vaccination is consistent with a survival study in guinea-pig conducted by Shang et al. (2012) in which the vaccination was shown to improve the effectiveness of combined therapy and prolong survival^13^. In the study, prior administration of BCG in adjunct to therapy was superior compared to therapy alone. An earlier study by Dhillon and Mitchison (1989) also assessed the impact of BCG vaccination in mice and guinea-pigs towards INH and RIF^12^. The study used an intravenous infection model of TB and chemotherapy was started soon after challenge. In the guinea-pig, prior BCG vaccination reduced the bacterial count in spleen after 20 days when administered in adjunct to INH. A similar trend was also observed with RIF notably during the first 14 days of the experiment, although the difference did not reach significance, similar to our findings using the *ex vivo* MGIA system.

With regard to INH, the drug is currently used as a prophylaxis treatment for individuals with latent TB, given for 9 months with an efficacy ranging from 60–90% for prevention of active TB^37^. A large observational prospective cohort study in Lima, Peru demonstrated the synergistic effect between historical BCG vaccination and INH prophylaxis, which was greater than each of the interventions alone in preventing active TB in household contacts of TB patients^38^. In the context of leprosy, historical BCG vaccination is known to boost the efficacy of RIF prophylaxis therapy. While RIF prophylaxis and historical BCG vaccination alone were shown to provide 58% and 57% protection against leprosy, the combination of both provided 80% protection in the study enrolling a large number of participants^14^. Findings in these studies suggest that there might be a beneficial effect of historical BCG vaccination on drug treatment which could have been underappreciated. We believe this is the first time the additive effect of immunoprophylaxis by routine BCG vaccination and chemoprophylaxis with INH was demonstrated using an *ex vivo* system and our results warrant further investigation in future epidemiological studies.

The mechanism of BCG-induced protection is thought to be via a CD4^+^ Th1 type response, with evidence showing that BCG-specific IFN-γ response measured with ELISpot was associated with reduced TB disease risk over the early years of life^31^. Nevertheless, there is emerging evidences that other T cell subsets (such as CD8^+^ and γδ T-cells) and NK cells may play a role in BCG-induced immune protection^23^. The guinea-pig study by Shang et al. suggested a possible mechanism for BCG enhancement of TB drug efficacy. In the animal study, combining BCG with drug therapy induced an increase in activated CD4^+^ T cells co-expressing CD45^hi^ and CT4^+^ as measured in blood, which was not observed in guinea-pigs receiving BCG alone^13^. In our study, although the *ex vivo* IFN-γ ELISPOT was not associated with growth inhibition, we observed increased IP-10 and, to a lesser extent, IFN-γ in the vaccinated group compared to the naïve, measured by ELISA, in the MGIA culture supernatant when PBMCs were co-cultured with INH. Moreover, IL-10 was associated with enhanced growth of mycobacteria *ex vivo* in the presence of drugs, although the impact appears to be independent from the vaccine effect. IFN-γ promotes macrophage activation by enhancing phagosomal maturation, inducing NO-dependent apoptosis, and modulating autophagy thus enhancing mycobacterial clearance^39^. While CD4^+^ T-cell is known to be a major source of IFN-γ, CD8^+^ T-cells, NK cells, γδ T-cells, and CD1-restricted T-cells also produce IFN-γ during infection with mycobacteria^40^.

We used flow-cytometry to identify the cellular source of IFN-γ in MGIA supernatants and identified the triple negative cells, which may represent NK cells, as the likely source of IFN-γ. The frequency of the CD3^−^ CD4^−^ CD8^−^ cells was increased in the BCG-vaccinated group during co-culture with 1 μg/ml of INH. Therefore, the putative NK cells may contribute to the increased IFN-γ production at this drug concentration. Recently, it was demonstrated that the production of IFN-γ and TNF-α by NK cells are functionally linked to their cytotoxic activities^41^. In our study, we saw increased frequencies of IFN-γ^+^ and TNF-α^+^ triple-negative CD3^−^ CD4^−^ CD8^−^ cells which were associated with the synergistic effect of historical BCG-vaccination and *ex vivo* drug-mediated killing of INH. We believe that historical BCG vaccination could enhance an NK cell response which contributes to *ex vivo* killing effect through the release of pro-inflammatory cytokines and cytotoxic granules.

Our findings are consistent with, and provide an explanation to, a recently published study by Jensen and colleagues (2017)^42^, in which a similar *ex vivo* MGIA system was used to assess protection from a TB vaccine candidate using a mouse model. While their study demonstrated a correlation between IFN-γ release and growth inhibition, the cellular source was not found among the investigated vaccine-specific T-cells, suggesting other cell populations such us the NK cells as a potential source. In a human study conducted in South Africa by the Scriba group, administration of BCG following isoniazid preventive therapy in latently-infected TB adults was associated with long-lived NK cells responses^43^. Evidence from immunological studies following immunisation with malaria and rabies vaccines have revealed the important role of NK cells in protection from vaccine-preventable diseases through their activation by antigen-specific CD4^+^ T cell-derived IL-2^44,45^. Moreover, a distinct subset of human NK cells expressing HLA-DR are known to expand in response to IL-2 and might aid immune responses to BCG^46^. In our study, we observe an increased frequency of IL-2^+^ CD4^+^ T-cells in the historically-vaccinated participants upon stimulation with BCG, suggesting that this cytokine could drive a BCG-specific enhancement of the NK cells responsible for improved *ex vivo* killing effect in the presence of INH.

In conclusion, we have demonstrated the synergistic effect between historical BCG vaccination and INH using an *ex vivo* system which support findings from previous observational and animal studies. Therapeutic vaccination aims to administer vaccine during TB treatment with the hope to improve treatment success and shorten duration of treatment^7^, and although several therapeutic TB vaccine candidates are available in the pipeline (reviewed in^47^) more are needed. Therefore, our MGIA platform offers an *ex vivo* assay that could help to identify and accelerate the development of candidate therapeutic TB vaccines using human PBMC samples, and its application for further larger investigations in clinical vaccine studies is warranted. Our preliminary data also suggest that the assay can be implemented in a murine model using splenocytes samples to assess vaccine impact on drug-mediated killing of mycobacteria. Our human study has signalled an association of putative NK cells and the combined effect between BCG vaccination and drug treatment, suggesting that these cells could be further explored as a target for novel therapeutic vaccine against TB.

## Methods

### Ethics statement

Participants were recruited under a protocol approved by the LSHTM Observational Research Ethics Committee (ref 8762). Written informed consent was obtained from all individuals prior to enrolment in the study. All procedures were in accordance with the Declaration of Helsinki, as agreed by the World Medical Association General Assembly (Washington, 2002) and ICH Good Clinical Practice (GCP).

### Study participants and blood sampling

This was an observational study in healthy adults with (i) no history of BCG vaccination or (ii) a history of BCG vaccination more than 6 months before study enrolment. Participants were aged 18 to 70 years with no evidence of exposure or infection with TB. Participants were excluded if they were suffering from any persistent medical condition or infection. Sample size was calculated based on the assumption of effect size 0.75, with power 0.8 and significance level 0.05 (participants per group = 29). Peripheral blood was collected at the amount of 50 ml and processed within 6 hours. Blood samples were collected in tubes containing sodium heparin (Sigma-Aldrich, Dorset, UK).

### PBMCs isolation and IFN-γ ELISpot

Peripheral blood mononuclear cells were isolated from heparinized whole blood by centrifugation over 15 ml LymphoPrep (Stemcell, Cambridge, UK) in a LeucoSep tube (Greiner Bio-One, Stonehouse, UK) according to the manufacturer’s instruction. PBMCs were cryopreserved in FBS (Labtech International Ltd, Uckfield, UK) containing 10% DMSO (Sigma-Aldrich) and stored in −80 °C freezer using CoolCell containers (VWR International, Lutterworth, UK). PBMCs were thawed and an *ex vivo* IFN-γ ELISpot assay was performed to assess antigen-specific responses as previously described^22^. In brief, PBMCs were incubated overnight for 18 hours with 20 μg/ml purified protein derivative (PPD) (Oxford Biosystem, Oxfordshire, UK). Positive control Phytohemagglutinin (PHA) (10 µg/ml, Sigma-Aldrich) and negative control (medium-only) wells were included for each participant samples.

### *Ex vivo* Mycobacterial Growth Inhibition Assay (MGIA)

Cryopreserved PBMCs were thawed and rested for 2 hours at 37 °C in antibiotic-free medium [RPMI-1640 (Sigma-Aldrich) +10% pooled human AB serum (Sigma-Aldrich) +2 mM L-Glutamine (Fisher Scientific, Loughborough, UK)] containing 10 U/ml benzonase (Insight Biotechnology, Wembley, UK). After the rest, the cells were counted, washed and re-suspended in the above-mentioned medium without benzonase. The percent viability of recovered cells was around 70 to 90% per vial. A 2-ml screw-cap tube containing 3 × 10^6^ PBMCs in 600 μl of medium was co-cultured with ∼100 Colony Forming Units of BCG for 4 days on a 360° rotator (VWR International, UK) at 37 °C. BCG Pasteur Aeras strain was obtained from Aeras (Rockville, MD, USA) and used as the immune target in the MGIA. In order to assess the potential synergistic effect of historical BCG vaccination and *ex vivo* drug-mediated killing of mycobacteria, 6 μl of drug at different concentrations was added in the MGIA system to the sample replicates from each participants. The drug final concentrations on the co-culture system were 1; 0.1 and 0.01 μg/ml for INH and 0.5; 0.1 and 0.01 μg/ml for RIF respectively. A control tube without drug was also set-up for each samples. INH and RIF were obtained from Sigma-Aldrich, UK and stock solutions were prepared in sterile tissue culture grade water and DMSO respectively as per manufacturer’s instruction.

After 4 days, the 2-ml tubes were centrifuged at 12,000 rpm for 10 minutes. The MGIA supernatants (500 µl) were transferred to other 2 ml tubes and frozen at −80 °C for further analysis. The remaining cells were then lysed by addition of 400 µl of sterile tissue culture grade water and vortexed 3 times with 5-minutes intervals. Lysate containing mycobacteria was transferred to a Bactec MGIT tube supplemented with PANTA antibiotics and oleic acid-albumin-dextrose-catalase (OADC) enrichment broth (all from Becton Dickinson, Oxford, UK). The tube was placed in a Bactec MGIT 960 and incubated until registered positive (measured as time to positivity [TTP]). Use of a standard curve enables conversion of the TTP of a sample tube into bacterial numbers (log CFU) (Supplementary Fig. S1). All work with cells pre-BCG infection and involving BCG infected samples was done in Biosafety Level (BSL) 2.

### ELISA

MGIA supernatants were analysed to assess cytokine concentrations by enzyme-linked immunosorbent assay (ELISA). The levels of following cytokines were measured: interferon-gamma (IFN-γ), tumor necrosis factor alpha (TNF-α), interleukin (IL)−12p40, IL-10, IL-17, IL-6, granulocyte-macrophage colony-stimulating factor (GM-CSF) and interferon-gamma-induced protein 10 (IP-10). The concentrations of IFN-γ, IL-12p40 and IL-6 were measured using BD OptiEIA kits (Becton Dickinson, UK), while TNF-α, GM-CSF and IP-10 DuoSet ELISA kits were obtained from R&D Systems (Abingdon, UK), and IL-10 ELISA MAX Standard and IL-17 ELISA MAX Deluxe from BioLegend (London, UK). Assays were performed as described by the manufacturers.

### Intracellular cytokine staining (ICS) assay and flow cytometry

PBMCs were thawed and rested for 2 hours in a 37 °C incubator with 5% CO_2_ after addition of 10 U/ml of benzonase. PBMCs were then incubated alone (medium only as negative control), with 5 μg/ml *Staphylococcus* enterotoxin B (SEB; Sigma, UK) as a positive control and with ∼100 CFU BCG (as per the MGIA protocol, with and without 1 μg/ml of INH). Incubation with BCG was performed for 4 days and the addition of SEB was performed on Day 3. Two hours after the addition of SEB to the positive control tubes, brefeldin A (Sigma, UK) was added to all tubes which were then incubated for 18 hours at 37 °C until day-4.

Following incubation, cells were washed with FACS buffer (PBS + 0.1% BSA + 0.01% sodium azide) and stained with Vivid live/dead reagent (Invitrogen) for 10 minutes at 4 °C in the dark. Cells were then surface stained with anti-CD4-APC (BD Biosciences), anti-CD19-efluor450 and anti-CD14-efluor450 (eBiosciences) for 30 minutes at 4 °C in the dark. After washing with FACS buffer, cells were permeabilised with Cytofix/Cytoperm reagent (BD Biosciences) at 4 °C for 20 min, washed in Perm Wash buffer (BD Biosciences) and stained with anti-CD3-Horizon-BV510, anti-IL-2-FITC, anti-TNFα-PE-Cy7 (BD Biosciences), anti-CD8-PE (eBiosciences) and anti-IFNγ-PerCPCy5.5 (Biolegend) for 30 min at room temperature in the dark. Cells were finally resuspended in 250 µL 1% paraformaldehyde (Sigma, UK) and filtered prior to acquisition.

Data was acquired using an LSRII flow cytometer (BD Biosciences) and FACSDiva acquisition software (BD Biosciences). Compensation was performed using tubes of OneComp eBeads (ThermoFisher, UK) individually stained with each fluorophor and compensation matrices were calculated with FACSDiva. ICS flow cytometry data was analysed using FlowJo software version 10 (TreeStar Inc., Ashland, OR, USA). Samples were gated sequentially on singlet, live, CD14^−^CD19^−^, lymphoid, CD3^+^, CD4^+^, CD8^+^, DN, TN cells and negative control stimulation tubes were used to set cytokine gates (see Supplementary Fig. S3).

Median cytokine responses in negative control tubes, as a percentage of the gated CD4^+^ T-cell population, were as follows: IFN-γ – 0.07%; IL-2–0.09%; TNF-α – 0.40%. Median cytokine responses in positive control tubes (SEB-stimulated) were as follows: IFN-γ – 3.16%; IL-2–4.24%; TNF-α – 24.35%. See Supplementary Table S2 for median cytokine responses of the gated CD8^+^ and DN T-cells as well as TN cells. Cytokine responses reported for all stimuli are after subtraction of background values measured in un-stimulated tubes. The median number of cellular events acquired for all tubes was 168,591 (IQR: 103871–225963).

### Statistical analysis

Statistical analyses were performed in Graphad Prism 7 (GraphPad, La Jolla, CA, USA). To identify statistical significance of *ex vivo* growth inhibition (log CFU values) and ELISA responses, one-way analysis of variance (ANOVA) and students *t*-test were used. Mann-Whitney *U* Test was performed to identify significant differences of the ELISpot and ICS responses between groups. Spearman’s correlation coefficient was used to test for correlations between growth inhibition and immune responses.

## Supplementary information

Supplementary Information
