# Supplementary material for: Historical BCG vaccination combined with drug treatment enhances inhibition of mycobacterial growth ex vivo in human peripheral blood cells
Source: Sci Rep. 2019 Mar 19;9:4842. doi: 10.1038/s41598-019-41008-4 (PMC6425030; doi:10.1038/s41598-019-41008-4)
Supplement: Supplementary file 1 — Supplementary Information [file 41598_2019_41008_MOESM1_ESM.docx]

**Supplementary Information**

**Historical BCG vaccination combined with drug treatment enhances inhibition of mycobacterial growth *ex vivo* in human peripheral blood cells**

Authors: Satria A. Prabowo^a,b^, Andrea Zelmer^a,b^, Lisa Stockdale^a,b^, Utkarsh Ojha^c^, Steven G. Smith^a,b^, Karin Seifert^a^, Helen A. Fletcher^a,b*^

^a^ Department of Immunology and Infection, Faculty of Infectious and Tropical Diseases, London School of Hygiene and Tropical Medicine, UK

^b^ Tuberculosis Centre, London School of Hygiene and Tropical Medicine, UK

^c^ Faculty of Medicine, Imperial College School of Medicine, Imperial College London, London, UK

*corresponding author: [helen.fletcher@lshtm.ac.uk](mailto:helen.fletcher@lshtm.ac.uk)


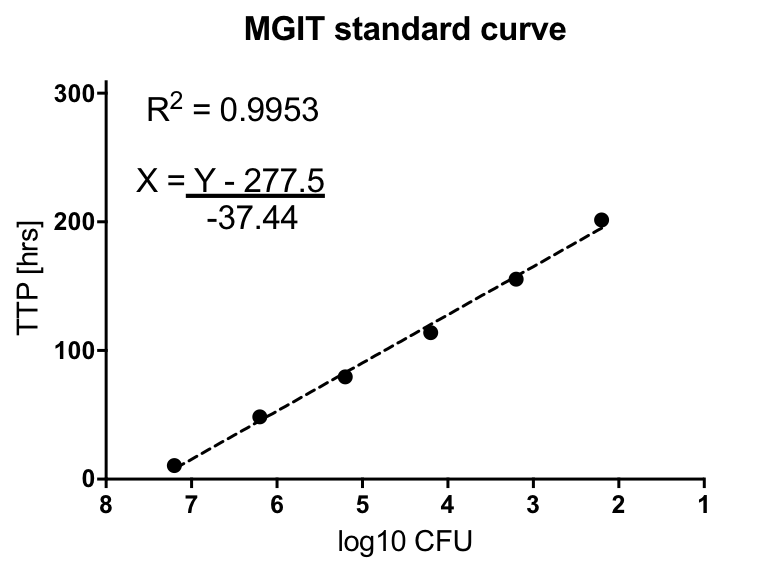


**Supplementary Figure S1. Standard curve of BCG Pasteur Aeras used to convert TTP to CFU.** A titration experiment was conducted to establish the relationship between log10 CFU and MGIT time to positivity (TTP). Linear regression analysis was carried out in GraphPad Prism. The resulting equation was used to calculate log10 CFU.

**
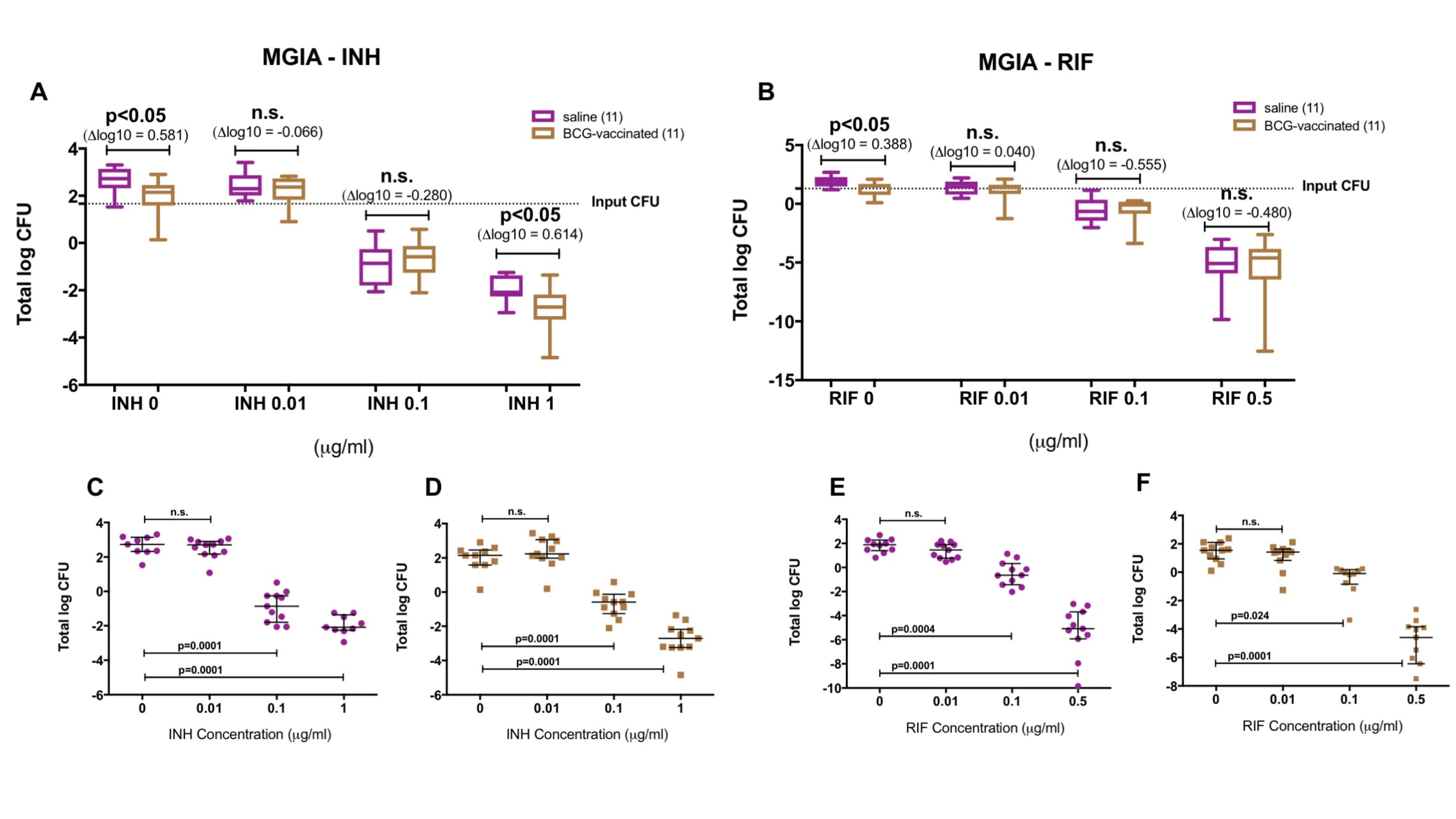
**

**Supplementary Figure S2. Growth inhibition in mice in the absence and presence of INH (A) and RIF (B).** C57Bl/6 mice were vaccinated with BCG Pasteur Aeras and sacrificed at week 6 for MGIA as previously described (Zelmer et al. BMC Infectious Diseases (2016) 16:412). MGIA was performed with 5 x 10^6^ mouse splenocytes, with ~100 BCG input and TB drugs as done with human PBMCs. Mycobacterial growth was assessed in titration curves. INH inhibited mycobacterial growth in a dose-dependent manner in the naïve and vaccinated groups (C and D), as well as RIF (E and F). Data from both groups was compiled in dose-response box plots to identify the BCG effect in addition to the INH- and RIF-mediated killing (A and B). Dots and squares in the titration curves (C – F) represent individual data points from the participants and the central lines indicate the median response with IQR. Each group is represented in a single box plot with range in the dose-response analysis (A and B). Data is pooled from two different experiments (n=11). Statistical significances were tested using one-way ANOVA (C – F) and unpaired t-test (A and B).

**
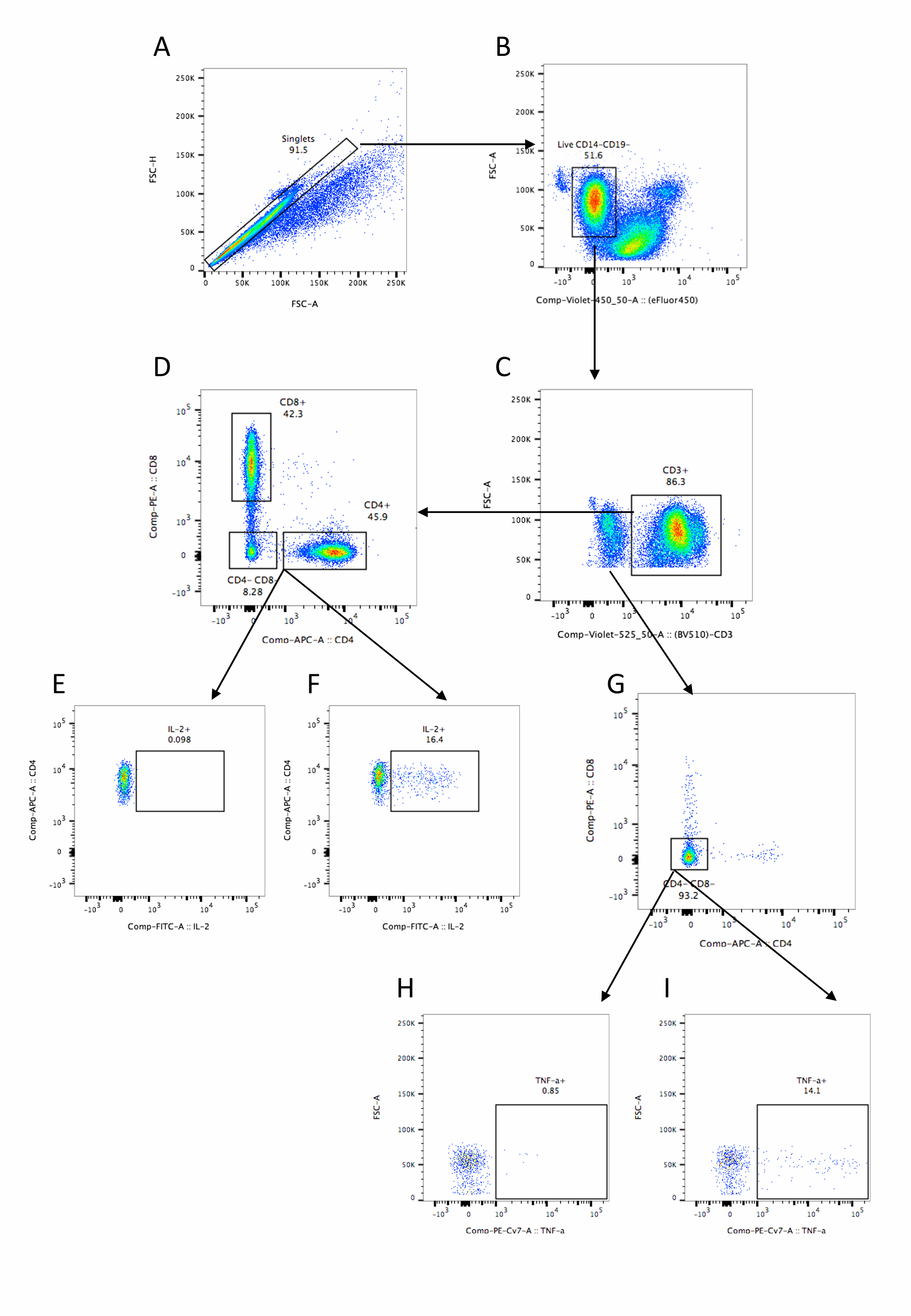
**

**Supplementary Figure S3. ICS Flow cytometry gating strategy.** Gating of singlet (A), dump negative (live, CD14-, CD19-) (B), CD3^+^ lymphocytes (C) and CD4^+^, CD8^+^ as well as CD4^-^ CD8^-^ T-cells (D) was performed in sequence for each sample. Furthermore, CD3^-^ CD4^-^ CD8^-^ lymphocytes (NK cells) was also gated (G). Cytokine gates were then set on unstimulated tubes (E and H) and copied to stimulated tubes (F and I). Gating for the following cytokines were set: IFN-γ, TNF-α and IL-2, with E & F and H & I represent examples for IL-2 and TNF-α, respectively.

**Supplementary Table S1. Summary of mean cytokine responses measured with ELISA assays, assessed from MGIA supernatant samples from the co-culture with INH and RIF.** Responses between BCG-naïve and BCG-vaccinated groups were compared using unpaired *t*-test. Correlations were investigated using Spearman’s correlation at certain drug concentrations (INH 1 µg/ml; RIF 0.01 and 0.1 µg/ml) based on the MGIA data. For the correlations, non-responders were excluded, defined as responses below the following cut-off of the ELISA assays: 7.5 pg/ml (IFN-γ), 5 pg/ml (IP-10), 3.5 pg/ml (IL-10), 20 pg/ml (TNF-α), 8 pg/ml (IL-12p40), 1 pg/ml (GM-CSF), 10 pg/ml (IL-6) and 0 pg/ml (IL-17). ND, not detected.

| **Cytokines** | **Mean Cytokine Response (pg/ml)** | | | | | | | | | | | | | | | |
| --- | --- | --- | --- | --- | --- | --- | --- | --- | --- | --- | --- | --- | --- | --- | --- | --- |
|  | **INH** | | | | | | | | **RIF** | | | | | | | |
|  | **Without drug** | | **0.01 μg/ml** | | **0.1 μg/ml** | | **1 μg/ml** | | **Without drug** | | **0.01 μg/ml** | | **0.1 μg/ml** | | **0.5 μg/ml** | |
|  | **Naïve** | **BCG** | **Naïve** | **BCG** | **Naïve** | **BCG** | **Naïve** | **BCG** | **Naïve** | **BCG** | **Naïve** | **BCG** | **Naïve** | **BCG** | **Naïve** | **BCG** |
| IFN-γ | 15.07 | 23.53 | 17.12 | 51.18 | 26.1 | 39.46 | 50.38 | 102.7 | 15.84 | 25.8 | 44.49 | 48.63 | 103.5 | 111.7 | 74.69 | 125.4 |
|  | p=0.177 | | p=0.141 | | p=0.289 | | **p=0.058** | | p=0.163 | | p=0.815 | | p=0.809 | | p=0.191 | |
| Correlation with MGIA |  | |  | |  | | **r -0.30**  ***p=0.049** | |  | | r 0.051  p=0.813 | | r -0.021  p=0.896 | |  | |
| IP-10 | 151.7 | 302.7 | 87.51 | 264.7 | 44.45 | 267.5 | 64.9 | 270.2 | 151.7 | 302.7 | 149 | 368.6 | 33.01 | 245.8 | 38.86 | 300.5 |
|  | p=0.151 | | **p=0.064** | | ***p=0.034** | | ***p=0.031** | | p=0.151 | | p=0.195 | | ***p=0.0055** | | ***p=0.035** | |
| Correlation with MGIA |  | |  | |  | | r -0.21  p=0.343 | |  | | r -0.34  p=0.180 | | r -0.26  p=0.258 | |  | |
| IL-10 | 35.31 | 62.44 | 42.54 | 76.05 | 43.62 | 75.86 | 46.65 | 53.1 | 35.31 | 62.44 | 37.83 | 49.3 | 37.18 | 62.66 | 51.75 | 69.38 |
|  | p=0.184 | | p=0.310 | | p=0.236 | | p=0.772 | | p=0.184 | | p=0.676 | | p=0.284 | | p=0.615 | |
| Correlation with MGIA |  | |  | |  | | **r 0.33**  ***p=0.033** | |  | | **r 0.34**  **p=0.087** | | **r 0.37**  ***p=0.019** | |  | |
| TNF-α | 10.97 | 82.04 | 25.6 | 113.9 | 17.91 | 132.2 | 23.87 | 43.82 | 10.97 | 82.04 | 22.82 | 49.68 | 17.61 | 81.85 | 65.19 | 129.2 |
|  | p=0.156 | | p=0.199 | | p=0.102 | | p=0.499 | | p=0.156 | | p=0.610 | | p=0.274 | | p=0.510 | |
| Correlation with MGIA |  | |  | |  | | r -0.22  p=0.529 | |  | | r 0.60  p=0.350 | | r 0.53  p=0.098 | |  | |
| IL-12p40 | 5.588 | 47.71 | 33.56 | 111.4 | 76.3 | 120.9 | 30.42 | 32.98 | 5.588 | 47.71 | 22.35 | 33.07 | 81.68 | 92.55 | 42.45 | 139.5 |
|  | p=0.179 | | p=0.169 | | p=0.602 | | p=0.923 | | p=0.179 | | p=0.722 | | p=0.824 | | p=0.324 | |
| Correlation with MGIA |  | |  | |  | | r -0.30  p=0.407 | |  | | r 0.64  p=0.139 | | r 0.44  p=0.075 | |  | |
| GM-CSF | 0.024 | 45.11 | 0 | 44.08 | 15.75 | 89.94 | 7.536 | 9.88 | 0.024 | 45.11 | 0.9781 | 8.111 | 5.658 | 35.87 | 4.117 | 57.44 |
|  | p=0.126 | | p=0.139 | | p=0.241 | | p=0.803 | | p=0.126 | | p=0.427 | | p=0.311 | | p=0.331 | |
| Correlation with MGIA |  | |  | |  | | r 0.080  p=0.333 | |  | | r -  p= - | | r -0.058  p=0.933 | |  | |
| IL-6 | 250.4 | 335.3 | 289 | 227.4 | 203.3 | 299.7 | 233.6 | 238.4 | 250.4 | 335.3 | 375.8 | 189.9 | 277.5 | 264.1 | 272.1 | 267.1 |
|  | p=0.324 | | p=0.558 | | p=0.274 | | p=0.954 | | p=0.324 | | p=0.139 | | p=0.886 | | p=0.965 | |
| Correlation with MGIA |  | |  | |  | | r 0.021  p=0.911 | |  | | r 0.22  p=0.424 | | r 0.059  p=0.759 | |  | |
| IL-17 | ND | ND | ND | ND | ND | ND | ND | ND | ND | ND | ND | ND | ND | ND | ND | ND |
|  | p= - | | p= - | | p= - | | p= - | | p= - | | p= - | | p= - | | p= - | |
| Correlation with MGIA |  | |  | |  | | r -  p= - | |  | | r -  p= - | | r -  p= - | |  | |

**Supplementary Table S2. Median cytokine responses in negative and positive control tubes of the ICS assay**

| **Gated cell population** | **Negative Control** | | | **Positive Control**  **(SEB-stimulated)** | | |
| --- | --- | --- | --- | --- | --- | --- |
|  | **IFN-γ** | **IL-2** | **TNF-α** | **IFN-γ** | **IL-2** | **TNF-α** |
| CD4^+^ T-cell | 0.07% | 0.09% | 0.40% | 3.16% | 4.24% | 24.35% |
| CD8^+^ T-cell | 0.19% | 0.05% | 0.27% | 11.55% | 1.88% | 23.65% |
| CD3^+^ CD4^-^ CD8^-^ (DN) T-cell | 0.63% | 1.47% | 0.80% | 0.20% | 0.09% | 1.05% |
| CD3^-^ CD4^-^ CD8^-^ (TN) cell | 0.12% | 0.10% | 0.85% | 2.10% | 1.80% | 4.68% |
